# Noninvasive *In-Vivo* Quantification of Mechanical Heterogeneity of Invasive Breast Carcinomas

**DOI:** 10.1371/journal.pone.0130258

**Published:** 2015-07-08

**Authors:** Tengxiao Liu, Olalekan A. Babaniyi, Timothy J. Hall, Paul E. Barbone, Assad A. Oberai

**Affiliations:** 1 Scientific Computation Research Center, Rensselaer Polytechnic Institute, Troy, NY, USA; 2 Mechanical Engineering, Boston University, Boston, MA, USA; 3 Medical Physics, University of Wisconsin, Madison, WI, USA; Beatson Institute for Cancer Research Glasgow, UNITED KINGDOM

## Abstract

Heterogeneity is a hallmark of cancer whether one considers the genotype of cancerous cells, the composition of their microenvironment, the distribution of blood and lymphatic microvasculature, or the spatial distribution of the desmoplastic reaction. It is logical to expect that this heterogeneity in tumor microenvironment will lead to spatial heterogeneity in its mechanical properties. In this study we seek to quantify the mechanical heterogeneity within malignant and benign tumors using ultrasound based elasticity imaging. By creating *in-vivo* elastic modulus images for ten human subjects with breast tumors, we show that Young’s modulus distribution in cancerous breast tumors is more heterogeneous when compared with tumors that are not malignant, and that this signature may be used to distinguish malignant breast tumors. Our results complement the view of cancer as a heterogeneous disease on multiple length scales by demonstrating that mechanical properties within cancerous tumors are also spatially heterogeneous.

## Introduction

**Fig 1:**
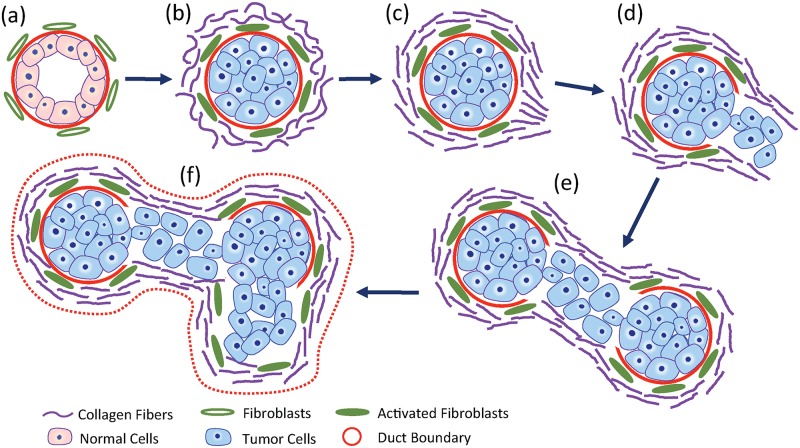
Schematic diagram of tumorigenesis in breast cancer (adapted from [7, 8]). (a) Healthy milk duct. (b) Proliferation of tumor cells within the duct is accompanied by desmoplasia in the extra-cellular matrix. (c) Changes in the morphology collagen fiber bundles from a wavy and tortuous state to a straight and taut state, and the emergence of a site where the fiber orientation is predominantly radial with respect to the tumor boundary. (d) Invasion of cancer cells to surrounding glandular tissue from this site. (e) Invasion of the cancer cells to nearby ducts. (f) A fully invasive tumor state. The dashed red curve represents the envelope of the tumor components that would appear as a region with elevated Young’s modulus.

Cancerous tumors in the breast often present as stiff lumps or lesions. One of the leading causes for the increased stiffness of these tumors is stromal desmoplasia, a process that leads to the proliferation of activated fibroblasts and myofibroblasts and the formation of a dense, collagen-rich stroma around the tumor [1, 2]. The increased collagen in turn leads to a stiffer mechanical response of the stroma. Desmoplasia is also thought to mechanically confine the tumor causing it to grow along very specific directions (see Fig 1). In particular, it has been observed that cancerous cells tend to migrate from a milk duct into the surrounding glandular tissue at locations where the collagen fiber alignment departs from a circumferential orientation to a radial orientation [3]. As a result the tumor invades the surrounding tissue and grows around these specific locations. This pattern of growth leads to the characteristic “stellate” or “spiculated” appearance of cancerous tumors [4]. It also leads to a spatially heterogeneous distribution of the stroma and, by extension, a heterogeneous distribution of tissue modulus. Other factors that contribute to the mechanical heterogeneity of the cancerous tumors include, functional heterogeneity in cancer-associated fibroblasts (CAFs) [5], which leads to heterogeneity in the degree of desmoplasia, and heterogeneity in the mechanical properties of tumor-associated vasculature and epithelium [6].

Elastography or elasticity imaging [9–11] refers to a variety of techniques to map the stiffness of tissue *in-vivo* and hence quantify the mechanical heterogeneity of tumors. These techniques rely on imaging tissue while it is mechanically deforming. Tissue motion may be either directly measured (e.g. phase contrast MR; c.f. [12]) or computed via image processing (e.g. cross correlation; c.f. [9, 11]. The observed tissue deformation, along with an appropriate mathematical model, is then used to estimate the mechanical properties.

**Fig 2:**
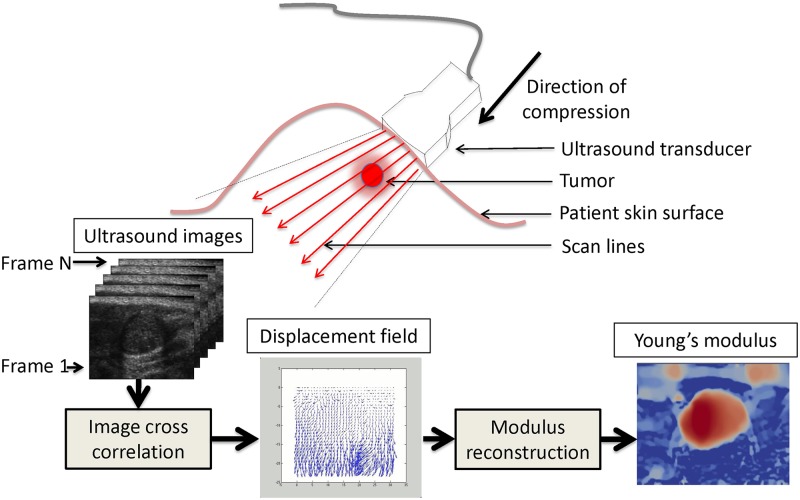
Schematic diagram of experimental setup: Ultrasound-based quasi-static elasticity imaging is a compression-based method to evaluate the mechanical properties of tissue *in-vivo*. First, an ultrasound transducer is gently pressed into the tissue, while acquiring a sequence of images. These images are used in a cross-correlation algorithm in order to determine the displacement field within the tissue. This deformation is then used in an inverse problem to determine the spatial distribution of elastic parameters.

Different approaches to elastography may be categorized by the type of imaging used to measure tissue motion, and the type of mechanical excitation used to cause tissue deformation. The present study was performed using quasi-static deformation that was measured via ultrasound. Fig 2 shows a schematic of the process. The essential features are as follows: An ultrasound transducer is held against the patient’s skin and provides real-time sequence of images. While imaging, a gentle compression is applied to the tissue. Cross correlation of sequential images provides a measurement of the displacement field within the tissue. Under conditions of small and relatively rapid (e.g. O(1Hz − 100Hz)) deformation, breast tissue is well modeled as isotropic incompressible linear elastic solid. In this case, the mechanical response may be completely characterized by a single material parameter, the Young’s modulus. The displacement field is then used to reconstruct the Young’s modulus distribution using inverse problem methods. For more details, please see Materials and Methods, below.

## Methods

We create *in-vivo* images of the Young’s modulus of breast tissue with benign and malignant tumors using ultrasound-based, quasi-static elasticity imaging at a resolution of about 200 microns. In this approach ultrasound images of the tumor *in-vivo* are acquired as the tissue is slowly compressed to about 10–12% strain. Thereafter, using image cross-correlation, the displacement field within this tissue is determined. Finally an inverse problem is solved to determine the spatial distribution of material parameters that is consistent with the observed displacements under the constraint of the equations of equilibrium.

The Radiofrequency (RF) data used in the study reported in this manuscript was collected for an earlier study conducted from 2002 to 2004. The patient data was anonymized and de-identified prior to analysis. Our use of this data was given full review and approval by the University of Wisconsin Health Sciences Institutional Review Board. Our study was also compliant with the Health Insurance Portability and Accountability Act.

In the earlier study, Radiofrequency (RF) echo data were recorded during *in-vivo* breast imaging at three institutions: the University of Kansas Medical Center in Kansas City, KS (approved by the KUMC Human Subjects Committee), Mayo Clinic in Rochester, MN (approved by the Mayo Foundation institutional review board), and the Charing Cross Hospital, London, UK (approved by the Riverside research ethics committee, Chelsea &Westminster Hospital (NHS Trust)). Informed consent was obtained from all enrolled patients.

The patient pool for the study comprised of ten patients with breast tumors selected from an earlier study [13]. Out of the ten tumors, five were benign fibroadenomas (FAs), and five were invasive ductal carcinomas (IDCs) as determined by percutaneous (core) or excisional biopsies.

Breast scanning was performed using Siemens SONOLINE Elegra ultrasound scanners fitted with high-frequency linear array transducers (either VFX135 or 7.5L40, Siemens Healthcare USA Inc., Mountain View, CA). Visual feedback to ensure the quality of radio-frequency echo data, and therefore the displacement data, was provided by a real-time strain imaging algorithm [14]. Displacement estimation between successive radio-frequency (RF) echo frames was performed with a modified block-matching algorithm that improves on the classical block-matching algorithm by constraining motion continuity using a dynamic programming technique and thereby reducing large tracking errors [15] (see supplementary videos for B-mode ultrasound movies and corresponding displacement movies). The size of each block was approximately 0.5*mm* × 1*mm*, and displacement estimates were first obtained on a 0.2*mm* × 0.2*mm* grid. These were then subsampled with quadratic precision onto a 0.12*mm* × 0.15*mm* grid and used for modulus inversion.

The spatial distribution of the elastic modulus was determined by minimizing the difference between the predicted and measured displacements, measured in a weighted *L*_2_ norm [16]. The predicted displacements were constrained to satisfy the equations of equilibrium for an incompressible linear elastic solid in a state of plane stress. The spatial distribution of the Young’s modulus was varied until the predicted displacements best matched the measured displacements. The predicted displacement and the Young’s modulus were both represented on the same 0.12*mm* × 0.15*mm* grid used for the measured displacements. The number of grid points was different for each tumor with an average of around 120 × 160 grid points. Total variation diminishing (TVD) regularization was used to provide robustness in the presence of noise in the measured displacements. The same regularization parameter was used for all tumors recognizing that the magnitude of noise in each case was the same. The resulting minimization algorithm was solved using a quasi-Newton algorithm [17] whose computational costs were reduced by using the solution of an adjoint elasticity problem [18]. The solution of each problem took about ten minutes on a typical desktop computer with four processors. Since no force measurements were available, the Young’s modulus distribution was determined up to an unknown multiplicative calibration factor.

## Results and Discussion

**Fig 3:**
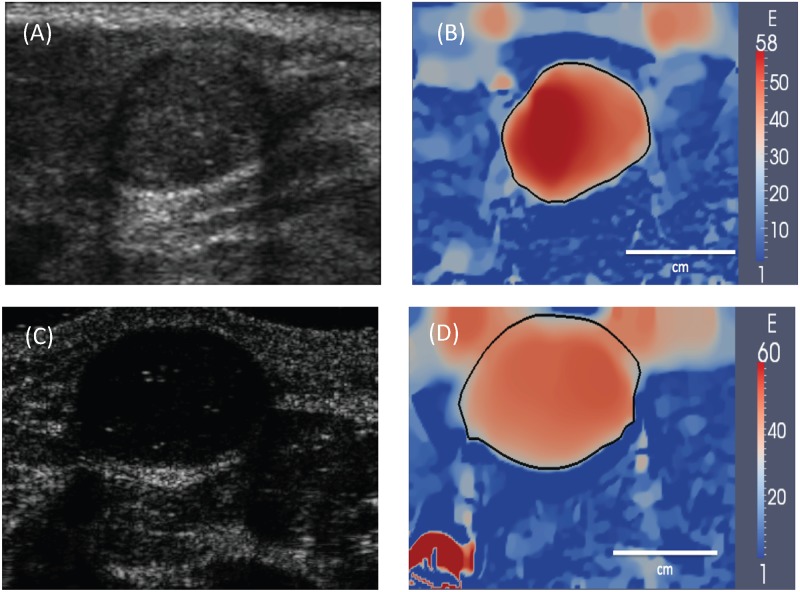
(A) and (C): B-mode ultrasound images of two typical fibroadenomas. (B) and (D): Corresponding Young’s modulus images generated using elasticity imaging. The tumor boundary is represented by a black curve that is drawn using 50 modulus value. The modulus distribution within the tumors is relatively homogeneous and the margins of the tumor are smooth.

**Fig 4:**
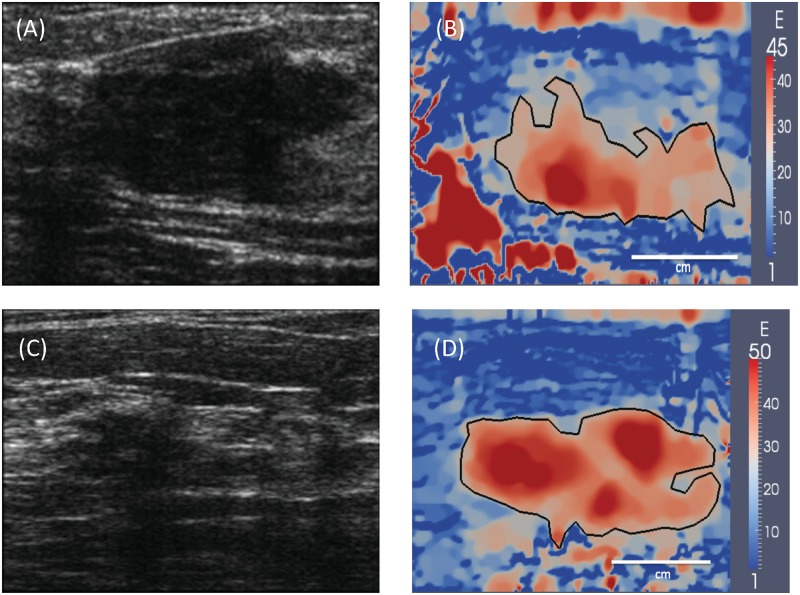
(A) and (C): B-mode ultrasound images of two typical invasive ductal carcinomas. (B) and (D): Corresponding Young’s modulus images generated using elasticity imaging. The tumor boundary is represented by a black curve that is drawn using 50 peak tumor modulus value. The modulus distribution within the tumors is heterogeneous and the margins of the tumors are rough.

The results for the distribution of the Young’s modulus are shown in Fig 3 for two typical fibroadenomas (FAs) and in Fig 4 for two typical invasive ductal carcinomas (IDCs). In these figures we have also shown the corresponding B-mode ultrasound images. First, we note that all the tumors are observed more clearly in the modulus images, where they stand out as regions of elevated stiffness. We also observe that modulus distribution within the cancerous tumors (IDCs) and the surrounding tissue is more heterogeneous when compared to benign tumors (FAs). Finally, we observe that the cancerous tumors display multiple foci of stiffness interspersed with softer regions.

For each of the ten patients the tumor margin was determined by using the 50% of peak tumor modulus contour in the modulus image and the B-mode image. Thereafter the heterogeneity of the modulus distribution within the tumor was quantified using a non-dimensional parameter that is approximately equal to the ratio of the tumor size to the correlation length of the modulus distribution within the tumor. This is parameter is given by,

$$\begin{array}{c} H\equiv \frac{1}{2}\sqrt{\frac{A\int_{\Omega }{\left|\nabla E\right|}^{2}d\Omega }{\int_{\Omega }E^{2}d\Omega }}, \end{array}$$

where Ω is the domain within the tumor, *A* is the area of Ω, and *E*(*x*) is the modulus map (see the Appendix for a derivation of *H*). When the correlation length of the modulus distribution is small compared to the size of the tumor, the tumor is mechanically heterogeneous, and the value of *H* is large.

**Fig 5:**
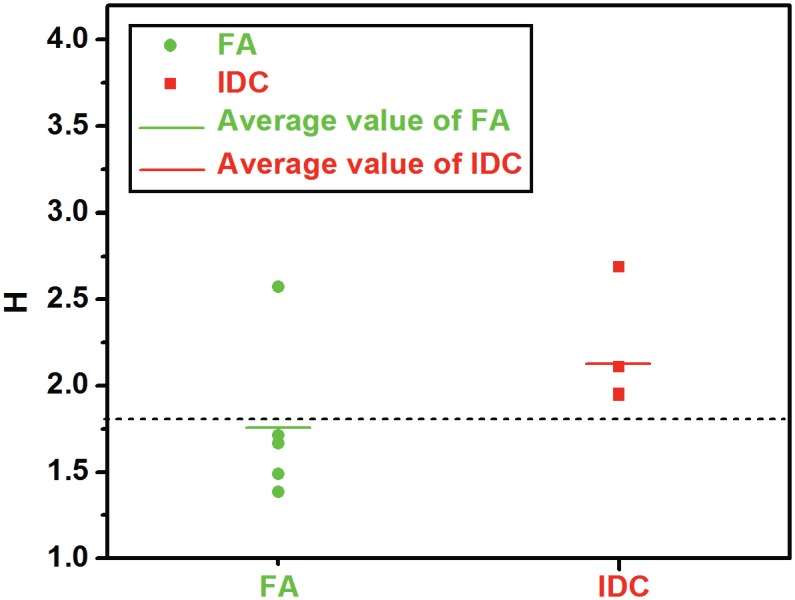
Value of the heterogeneity parameter for the five invasive ductal carcinomas (represented by red squares) and fibroadenomas (represented by green circles). The average value for the IDCs is 2.13 and the average value for the FAs is 1.76. Using H > 1.8 as a criterion for malignancy yields 90% diagnostic accuracy.

The value of this parameter for the five FAs and IDCs is presented in Fig 5. We note that when compared to the FAs, the value of *H* for the IDCs is large. This implies that heterogeneity at this scale is not generically associated with tissue stiffening, but is specific to malignancy. The value of *H* for benign lesions was 1.765 ± 0.420, and for malignant lesions it was 2.130 ± 0.285 (Mean ± standard deviation). The difference in the value of *H* between benign and malignant lesions was 0.365 with a 95% confidence interval of [-0.057, 0.788]. Since this interval includes zero, these results would not be considered statistically significant, even though the lower limit is quite close to zero. We attribute this to the one outlying fibroadenoma that has a large value of *H* (= 2.572). If this sample is removed from our set, then the average value of *H* for benign lesions becomes 1.563 ± 0.132 (Mean ± standard deviation), and the difference in the average value of *H* between benign and malignant lesions is 0.567 with a 95% confidence interval of [0.299, 0.835]. This result would be considered statistically significant. This sensitivity to the inclusion (or exclusion) of a single sample illustrates that though our results are promising, they need to be tested on a larger sample set in order to be considered statistically significant. If we use the value of *H* = 1.8 as a threshold of malignancy we could correctly classify 9 out of 10 lesions. These numbers compare favorably with the current diagnostic performance of mammography in conjunction with ultrasound [19].

Our results indicate that the distribution of stiffness within and around cancerous tumors is heterogeneous. This observation is consistent with recent results that suggest subjective observations of mechanical heterogeneity in tumors can improve the specificity (reduce false positives) of ultrasound in diagnosing breast cancer [20]. In that study the authors relied on shear-wave elastography to generate modulus images at a resolution of about 2*mm* × 2*mm*. In comparison we have used quasi-static elastography, and our resolution is about ten times finer. This allows us to compute a quantitative measure to the mechanical heterogeneity within the tumor. They are also consistent with recent observations of mechanical heterogeneity in an excised transgenic mammary mouse tumor model [6]. In this study the spatial variation of stiffness was determined at a resolution of 0.1*mm* × 0.1*mm* using atomic force microscopy (AFM) and it was observed that tumorigenesis led to significant mechanical heterogeneity. Taken together with results at the cellular level [5], stromal level [3], and microvasculature [6], these results round out the picture of mechanical heterogeneity of cancer across multiple length scales.

Based on the organization of collagen fibers (see Fig 1) malignant tumors would likely display anisotropic elastic behavior. Indeed, this has already been reported in some studies [21, 22]. However, when solving the inverse problem we have assumed that the tissue is isotropic. This raises the question whether in our reconstructions anisotropy has been misconstrued as heterogeneity. Based on or earlier results [23], we believe that this is not the case. In [23], we considered an anisotropic inclusion embedded in an isotropic medium, and first calculated its deformation in response to a load. Thereafter, we used this deformation in conjunction with an isotropic constitutive law, and reconstructed the spatial distribution of elastic parameters. That is we incorrectly assumed that the material was isotropic. We found that incorrect assumption lead to an error in the value of the reconstructed elastic parameters but did not introduce any spurious heterogeneity.

A cancerous tumor is comprised of several components that include cancerous epithelial cells, tumor microvasculature and the extra-cellular matrix. The resolution of our modulus images is such that we do not discern the stiffness of each individual component; rather, we create modulus images of an aggregate of these components. Thus the heterogeneity we observe may be attributed to the mechanical heterogeneity observed in these components, as discussed below.

**Extra-cellular matrix**. It is well known that desmoplastic reaction to cancer leads to the formation of a dense collagen-rich stroma around the tumor that confines the tumor. An increase in the concentration of collagen leads to an increase in the stiffness of tissue. As a result cancerous tumors are clearly seen as regions of elevated modulus in elastic modulus images. Further, at some specific sites in the stroma the alignment of the collagenous fibers is altered from a circumferential to a radial orientation and the tumor cells migrate and invade the surrounding tissue from these sites [3]. Consequently, the tumor and the stroma grow around these specific sites and the tumor attains a heterogeneous shape rather than a simple spherical form. This mechanism of growth (represented schematically in Fig 1) then implies that the tumor would appear as a heterogeneous region of elevated elastic modulus in a modulus image also. In addition to this, significant phenotypic and functional heterogeneity has been observed in cancer-associated fibroblasts (CAFs) [5] even within the same tumor. Since CAFs are associated with enhanced extra-cellular matrix production, it is reasonable to expect that their functional heterogeneity would lead to varying levels of stiffness within the same tumor.

**Tumor-associated vasculature**. Recent high-resolution atomic force microscopy (AFM) indentation experiments in live and snap-frozen fluorescently labeled mammary tissues in PyMT mice have indicated that there is significant variability in the stiffness of the vasculature within a tumor [6]. In particular the Young’s modulus of the vasculature in the tumor core is significantly larger than that of the vasculature at the invasive front of the tumor. This would contribute to the overall mechanical heterogeneity observed in our *in-vivo* modulus maps.

**Tumor-cells**. The same *in situ* AFM study [6] revealed that cancerous cells are significantly stiffer than their healthy counterparts, and that they contribute to the stiffness of the tumors. It was also observed that the mechanical properties of cells within the same tumor were significantly different. It was argued that this could be due to the spatial variation in the stress state within the tumor, which would cause cells in highly-stressed regions to be stiffer, or due to the inherent genetic heterogeneity of the cancer cells. In either case, this variation in the mechanical properties of cancerous epithelial cells will further contribute to the observed heterogeneity in the elastic modulus in our images.

## Conclusions

Heterogeneity has long been considered the hallmark of cancer. In this study, by creating high-resolution, *in-vivo* images of the Young’s modulus of breast tissue, we have demonstrated this heterogeneity also extends to the mechanical properties of cancerous tumors. Further, we have quantified this heterogeneity through a single parameter that is determined from these images and demonstrated that this parameter has the potential to non-invasively diagnose malignant tumors.

## Appendix

For a field *E*(*x*), the normalized autocorrelation function (denoted by *g*_*x*_(*r*)) along the unit vector *e*_*x*_, is defined as

$$g_{x}\left(r\right)=\frac{\left\langle E\left(x+re_{x}/2\right)E\left(x-re_{x}/2\right)\right\rangle }{\left\langle E^{2}\left(x\right)\right\rangle },$$ (1)

where the angular brackets denote an ensemble average. For fields that are assumed to be spatially homogeneous the ensemble average may be replaced by an area integral (in two dimensions). That is

$$g_{x}\left(r\right)=\frac{\int_{\Omega }E\left(x+re_{x}/2\right)E\left(x-re_{x}/2\right)dx}{\int_{\Omega }E^{2}\left(x\right)dx}.$$ (2)

Here Ω is the extent of the domain. Expanding the integrand in a Taylor’s series about *x*, and retaining terms that are *O*(|*r*|^2^), we arrive at

$$g_{x}\left(r\right)\approx \frac{\int_{\Omega }(E^{2}+\frac{r^{2}}{4}(-{\left(\frac{\partial E}{\partial x}\right)}^{2}+E\frac{\partial^{2}E}{\partial x^{2}}))dx}{\int_{\Omega }E^{2}dx}.$$ (3)

Performing integration by parts on the last term in the numerator, and neglecting the resulting boundary term (whose integrand may change signs along the boundary) in favor of the integrals over the domain (whose integrand are of the same sign over the entire domain), we obtain

$$g_{x}\left(r\right)\approx 1-\frac{r^{2}}{2}\frac{\int_{\Omega }{\left(\frac{\partial E}{\partial x}\right)}^{2}dx}{\int_{\Omega }E^{2}dx}.$$ (4)

Let *ρ*_*x*_ denote the distance at which the correlation vanishes, that is *g*_*x*_(*ρ*_*x*_) = 0. This condition yields,

$$\rho_{x}^{2}=\frac{2\int_{\Omega }E^{2}dx}{\int_{\Omega }{\left(\frac{\partial E}{\partial x}\right)}^{2}dx}.$$ (5)

Similarly along the *y*-direction, *ρ*_*y*_ is given by

$$\rho_{y}^{2}=\frac{2\int_{\Omega }E^{2}dx}{\int_{\Omega }{\left(\frac{\partial E}{\partial y}\right)}^{2}dx}.$$ (6)

The harmonic mean of the squares of these lengths, denoted by *ρ* ^2^ is

$$\rho^{2}=\frac{2}{\rho_{x}^{-2}+\rho_{y}^{-2}}=\frac{4\int_{\Omega }E^{2}dx}{\int_{\Omega }{\left|\nabla E\right|}^{2}dx}.$$ (7)

By its definition *ρ* is a measure of the correlation length of the spatial distribution of *E* (in turbulence, when applied to the velocity field, it is referred to as the Taylor microscale). Therefore an appropriate measure of the heterogeneity in this distribution is the ratio of the size of the domain ($\sqrt{A}$, where *A* is the area of Ω) to the correlation length *ρ*, that is

$$H=\frac{\sqrt{A}}{\rho }=\frac{1}{2}\sqrt{\frac{A\int_{\Omega }{\left|\nabla E\right|}^{2}dx}{\int_{\Omega }E^{2}dx}}.$$ (8)

## Supporting Information

S1 Video A sequence of b-mode ultrasound images for the fibroadenoma depicted in (A) and (B) in Fig 3. This sequence is acquired as the tissue is compressed to around 12% strain.
(MP4)

S2 Video A sequence of displacement vector images for the fibroadenoma depicted in (A) and (B) in Fig 3. This sequence is obtained by cross-correlating the radio-frequency ultrasound data acquired as the tissue is compressed to around 12% strain.
(MP4)

S3 Video A sequence of b-mode ultrasound images for the invasive ductal carcinoma depicted in (C) and (D) in Fig 4. This sequence is acquired as the tissue is compressed to around 12% strain.
(MP4)

S4 Video A sequence of displacement vector images for the invasive ductal carcinoma depicted in (C) and (D) in Fig 4. This sequence is obtained by cross-correlating the radio-frequency ultrasound data acquired as the tissue is compressed to around 12% strain.
(MP4)
